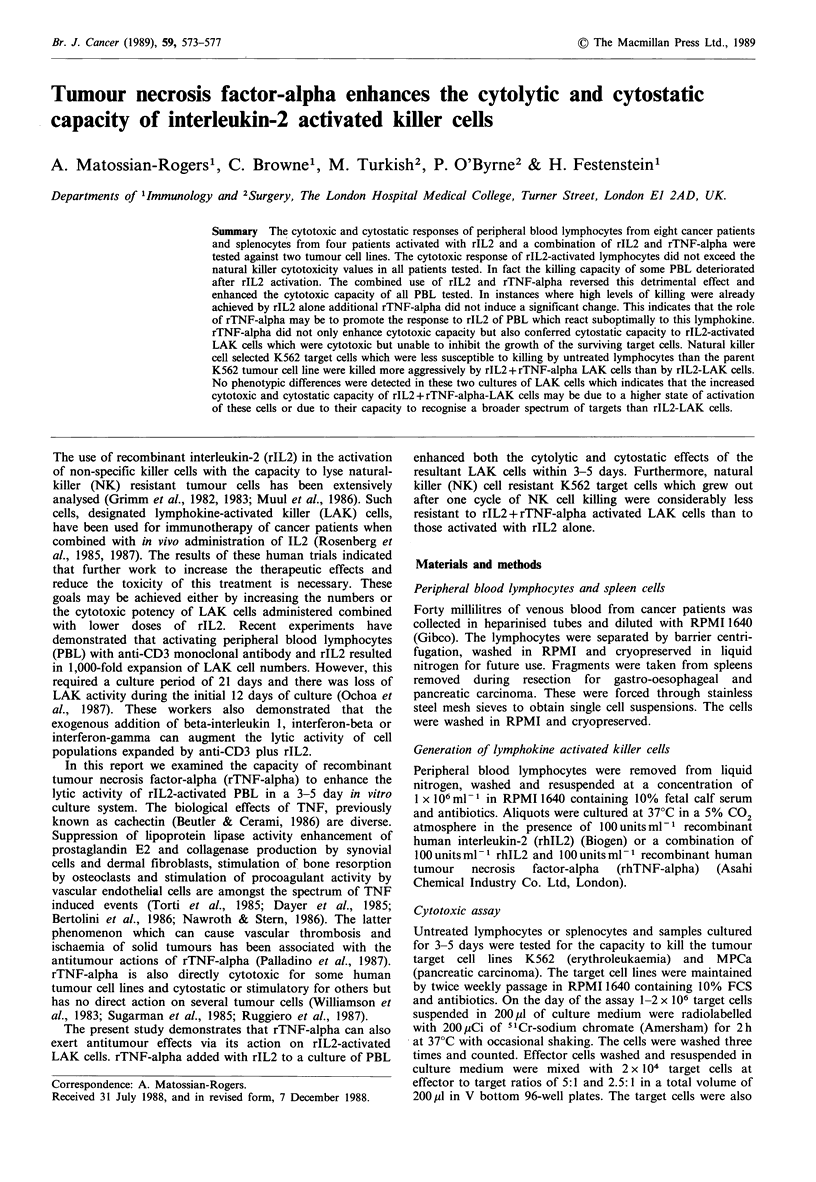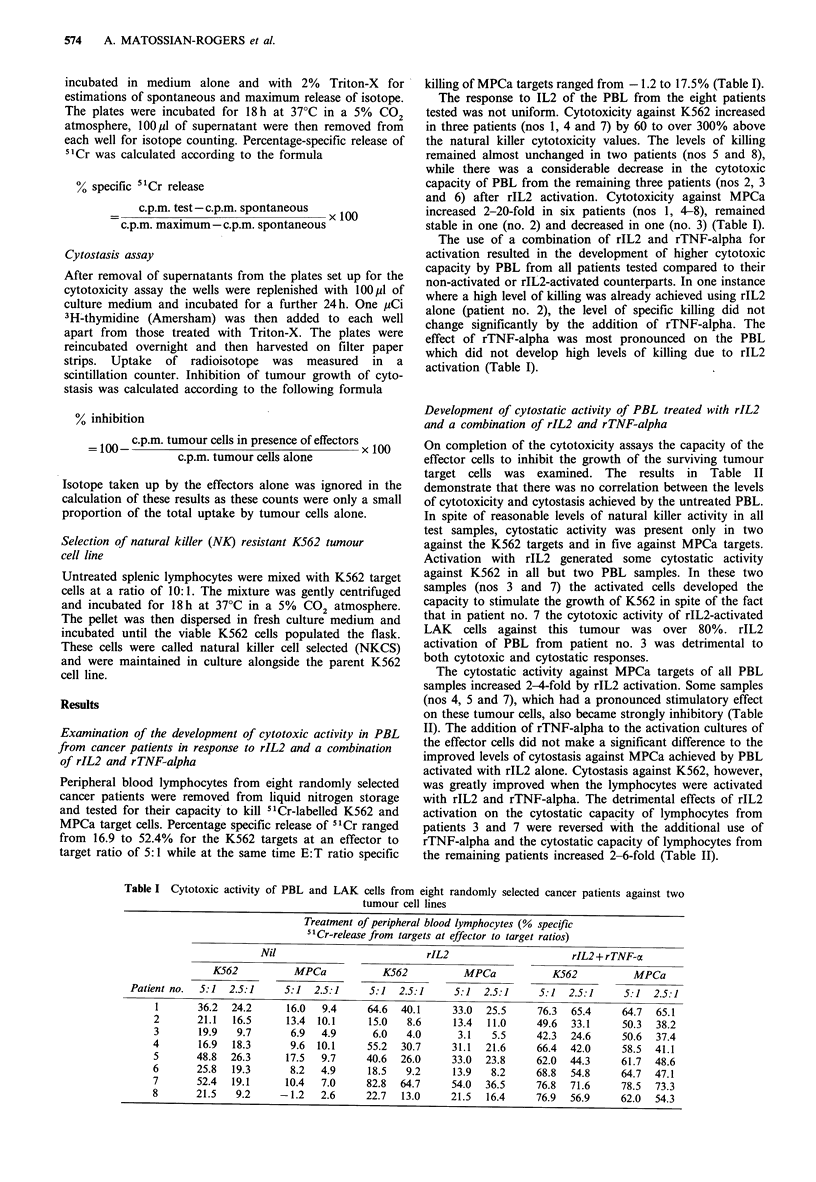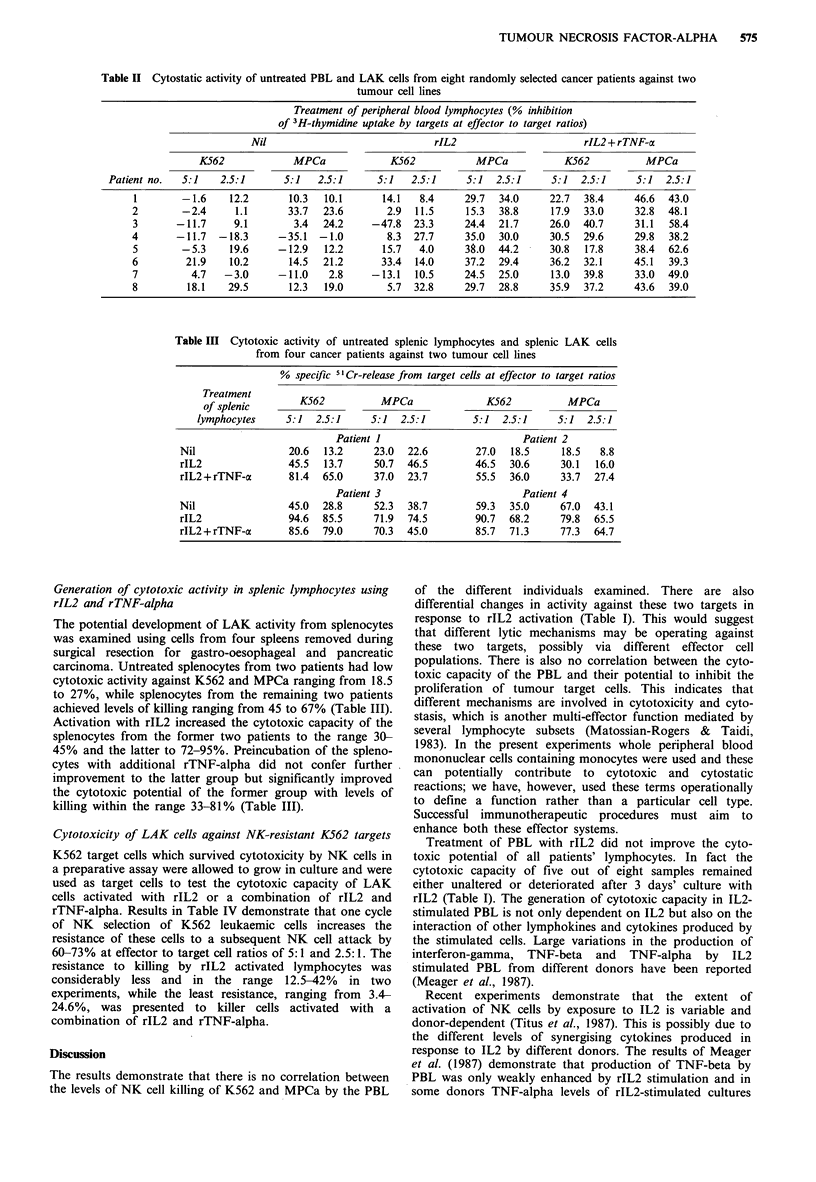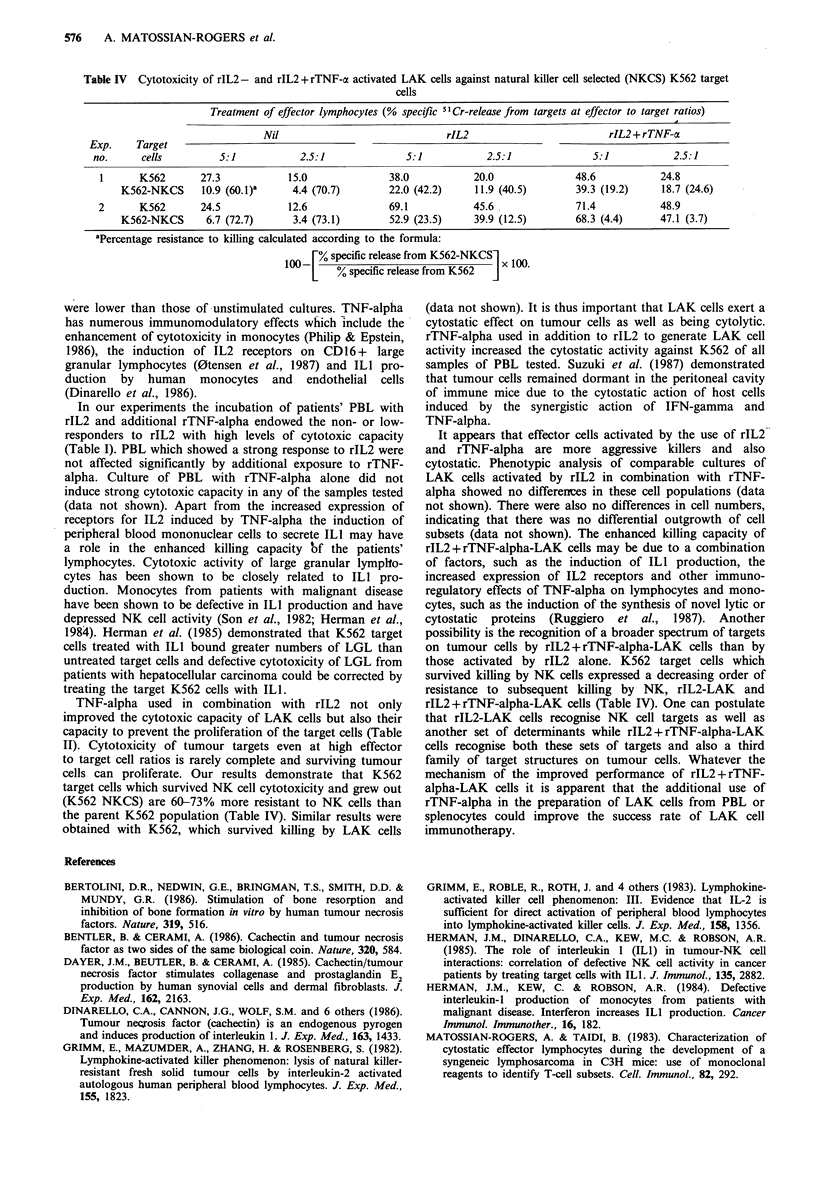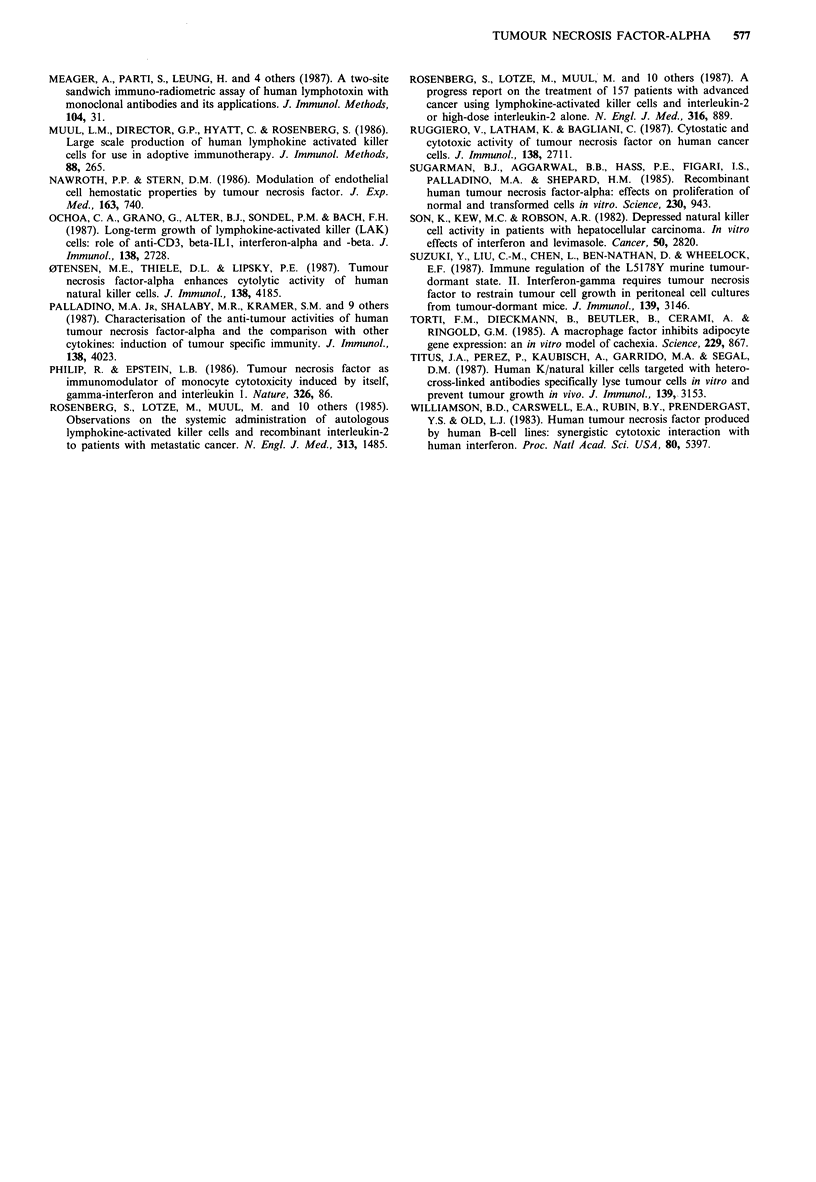# Tumour necrosis factor-alpha enhances the cytolytic and cytostatic capacity of interleukin-2 activated killer cells.

**DOI:** 10.1038/bjc.1989.116

**Published:** 1989-04

**Authors:** A. Matossian-Rogers, C. Browne, M. Turkish, P. O'Byrne, H. Festenstein

**Affiliations:** Department of Immunology, London Hospital Medical College, UK.

## Abstract

The cytotoxic and cytostatic responses of peripheral blood lymphocytes from eight cancer patients and splenocytes from four patients activated with rIL2 and a combination of rIL2 and rTNF-alpha were tested against two tumour cell lines. The cytotoxic response of rIL2-activated lymphocytes did not exceed the natural killer cytotoxicity values in all patients tested. In fact the killing capacity of some PBL deteriorated after rIL2 activation. The combined use of rIL2 and rTNF-alpha reversed this detrimental effect and enhanced the cytotoxic capacity of all PBL tested. In instances where high levels of killing were already achieved by rIL2 alone additional rTNF-alpha did not induce a significant change. This indicates that the role of rTNF-alpha may be to promote the response to rIL2 of PBL which react suboptimally to this lymphokine. rTNF-alpha did not only enhance cytotoxic capacity but also conferred cytostatic capacity to rIL2-activated LAK cells which were cytotoxic but unable to inhibit the growth of the surviving target cells. Natural killer cell selected K562 target cells which were less susceptible to killing by untreated lymphocytes than the parent K562 tumour cell line were killed more aggressively by rIL2 + rTNF-alpha LAK cells than by rIL2-LAK cells. No phenotypic differences were detected in these two cultures of LAK cells which indicates that the increased cytotoxic and cytostatic capacity of rIL2 + rTNF-alpha-LAK cells may be due to a higher state of activation of these cells or due to their capacity to recognise a broader spectrum of targets than rIL2-LAK cells.


*Br. J. Cancer* (1989), **59**, 573–577 © The Macmillan Press Ltd., 1989

### Tumour necrosis factor-alpha enhances the cytolytic and cytostatic capacity of interleukin-2 activated killer cells

A. Matossian-Rogers[1], C. Browne[1], M. Turkish[2], P. O'Byrne[2] & H. Festenstein[1]

*Departments of [1]Immunology and [2]Surgery, The London Hospital Medical College, Turner Street, London E1 2AD, UK.*

**Summary** The cytotoxic and cytostatic responses of peripheral blood lymphocytes from eight cancer patients and splenocytes from four patients activated with rIL2 and a combination of rIL2 and rTNF-alpha were tested against two tumour cell lines. The cytotoxic response of rIL2-activated lymphocytes did not exceed the natural killer cytotoxicity values in all patients tested. In fact the killing capacity of some PBL deteriorated after rIL2 activation. The combined use of rIL2 and rTNF-alpha reversed this detrimental effect and enhanced the cytotoxic capacity of all PBL tested. In instances where high levels of killing were already achieved by rIL2 alone additional rTNF-alpha did not induce a significant change. This indicates that the role of rTNF-alpha may be to promote the response to rIL2 of PBL which react suboptimally to this lymphokine. rTNF-alpha did not only enhance cytotoxic capacity but also conferred cytostatic capacity to rIL2-activated LAK cells which were cytotoxic but unable to inhibit the growth of the surviving target cells. Natural killer cell selected K562 target cells which were less susceptible to killing by untreated lymphocytes than the parent K562 tumour cell line were killed more aggressively by rIL2+rTNF-alpha LAK cells than by rIL2-LAK cells. No phenotypic differences were detected in these two cultures of LAK cells which indicates that the increased cytotoxic and cytostatic capacity of rIL2+rTNF-alpha-LAK cells may be due to a higher state of activation of these cells or due to their capacity to recognise a broader spectrum of targets than rIL2-LAK cells.

The use of recombinant interleukin-2 (rIL2) in the activation of non-specific killer cells with the capacity to lyse natural-killer (NK) resistant tumour cells has been extensively analysed (Grimm *et al.*, 1982, 1983; Muul *et al.*, 1986). Such cells, designated lymphokine-activated killer (LAK) cells, have been used for immunotherapy of cancer patients when combined with *in vivo* administration of IL2 (Rosenberg *et al.*, 1985, 1987). The results of these human trials indicated that further work to increase the therapeutic effects and reduce the toxicity of this treatment is necessary. These goals may be achieved either by increasing the numbers or the cytotoxic potency of LAK cells administered combined with lower doses of rIL2. Recent experiments have demonstrated that activating peripheral blood lymphocytes (PBL) with anti-CD3 monoclonal antibody and rIL2 resulted in 1,000-fold expansion of LAK cell numbers. However, this required a culture period of 21 days and there was loss of LAK activity during the initial 12 days of culture (Ochoa *et al.*, 1987). These workers also demonstrated that the exogenous addition of beta-interleukin 1, interferon-beta or interferon-gamma can augment the lytic activity of cell populations expanded by anti-CD3 plus rIL2.

In this report we examined the capacity of recombinant tumour necrosis factor-alpha (rTNF-alpha) to enhance the lytic activity of rIL2-activated PBL in a 3–5 day *in vitro* culture system. The biological effects of TNF, previously known as cachectin (Beutler & Cerami, 1986) are diverse. Suppression of lipoprotein lipase activity enhancement of prostaglandin E2 and collagenase production by synovial cells and dermal fibroblasts, stimulation of bone resorption by osteoclasts and stimulation of procoagulant activity by vascular endothelial cells are amongst the spectrum of TNF induced events (Torti *et al.*, 1985; Dayer *et al.*, 1985; Bertolini *et al.*, 1986; Nawroth & Stern, 1986). The latter phenomenon which can cause vascular thrombosis and ischaemia of solid tumours has been associated with the antitumour actions of rTNF-alpha (Palladino *et al.*, 1987). rTNF-alpha is also directly cytotoxic for some human tumour cell lines and cytostatic or stimulatory for others but has no direct action on several tumour cells (Williamson *et al.*, 1983; Sugarman *et al.*, 1985; Ruggiero *et al.*, 1987).

The present study demonstrates that rTNF-alpha can also exert antitumour effects via its action on rIL2-activated LAK cells. rTNF-alpha added with rIL2 to a culture of PBL enhanced both the cytolytic and cytostatic effects of the resultant LAK cells within 3–5 days. Furthermore, natural killer (NK) cell resistant K562 target cells which grew out after one cycle of NK cell killing were considerably less resistant to rIL2+rTNF-alpha activated LAK cells than to those activated with rIL2 alone.

### Materials and methods

### *Peripheral blood lymphocytes and spleen cells*

Forty millilitres of venous blood from cancer patients was collected in heparinised tubes and diluted with RPMI 1640 (Gibco). The lymphocytes were separated by barrier centrifugation, washed in RPMI and cryopreserved in liquid nitrogen for future use. Fragments were taken from spleens removed during resection for gastro-oesophageal and pancreatic carcinoma. These were forced through stainless steel mesh sieves to obtain single cell suspensions. The cells were washed in RPMI and cryopreserved.

### *Generation of lymphokine activated killer cells*

Peripheral blood lymphocytes were removed from liquid nitrogen, washed and resuspended at a concentration of $1\times10^{6}\,ml^{-1}$ in RPMI 1640 containing 10% fetal calf serum and antibiotics. Aliquots were cultured at 37°C in a 5% $CO_2$ atmosphere in the presence of $100\,units\,ml^{-1}$ recombinant human interleukin-2 (rhIL2) (Biogen) or a combination of $100\,units\,ml^{-1}$ rhIL2 and $100\,units\,ml^{-1}$ recombinant human tumour necrosis factor-alpha (rhTNF-alpha) (Asahi Chemical Industry Co. Ltd, London).

### *Cytotoxic assay*

Untreated lymphocytes or splenocytes and samples cultured for 3–5 days were tested for the capacity to kill the tumour target cell lines K562 (erythroleukaemia) and MPCa (pancreatic carcinoma). The target cell lines were maintained by twice weekly passage in RPMI 1640 containing 10% FCS and antibiotics. On the day of the assay $1-2\times10^{6}$ target cells suspended in 200 µl of culture medium were radiolabelled with 200 µCi of $^{51}Cr$-sodium chromate (Amersham) for 2 h at 37°C with occasional shaking. The cells were washed three times and counted. Effector cells washed and resuspended in culture medium were mixed with $2\times10^{4}$ target cells at effector to target ratios of 5:1 and 2.5:1 in a total volume of 200 µl in V bottom 96-well plates. The target cells were also

Correspondence: A. Matossian-Rogers.
Received 31 July 1988, and in revised form, 7 December 1988.

incubated in medium alone and with 2% Triton-X for estimations of spontaneous and maximum release of isotope. The plates were incubated for 18 h at 37°C in a 5% $CO_2$ atmosphere, 100 μl of supernatant were then removed from each well for isotope counting. Percentage-specific release of $^{51}$Cr was calculated according to the formula

$$\% \text{ specific } ^{51}\text{Cr release} = \frac{\text{c.p.m. test} - \text{c.p.m. spontaneous}}{\text{c.p.m. maximum} - \text{c.p.m. spontaneous}} \times 100$$

*Cytostasis assay*

After removal of supernatants from the plates set up for the cytotoxicity assay the wells were replenished with 100 μl of culture medium and incubated for a further 24 h. One μCi $^3$H-thymidine (Amersham) was then added to each well apart from those treated with Triton-X. The plates were reincubated overnight and then harvested on filter paper strips. Uptake of radioisotope was measured in a scintillation counter. Inhibition of tumour growth of cytostasis was calculated according to the following formula

$$\% \text{ inhibition} = 100 - \frac{\text{c.p.m. tumour cells in presence of effectors}}{\text{c.p.m. tumour cells alone}} \times 100$$

Isotope taken up by the effectors alone was ignored in the calculation of these results as these counts were only a small proportion of the total uptake by tumour cells alone.

*Selection of natural killer (NK) resistant K562 tumour cell line*

Untreated splenic lymphocytes were mixed with K562 target cells at a ratio of 10:1. The mixture was gently centrifuged and incubated for 18 h at 37°C in a 5% $CO_2$ atmosphere. The pellet was then dispersed in fresh culture medium and incubated until the viable K562 cells populated the flask. These cells were called natural killer cell selected (NKCS) and were maintained in culture alongside the parent K562 cell line.

### Results

*Examination of the development of cytotoxic activity in PBL from cancer patients in response to rIL2 and a combination of rIL2 and rTNF-alpha*

Peripheral blood lymphocytes from eight randomly selected cancer patients were removed from liquid nitrogen storage and tested for their capacity to kill $^{51}$Cr-labelled K562 and MPCa target cells. Percentage specific release of $^{51}$Cr ranged from 16.9 to 52.4% for the K562 targets at an effector to target ratio of 5:1 while at the same time E:T ratio specific killing of MPCa targets ranged from −1.2 to 17.5% (Table I).

The response to IL2 of the PBL from the eight patients tested was not uniform. Cytotoxicity against K562 increased in three patients (nos 1, 4 and 7) by 60 to over 300% above the natural killer cytotoxicity values. The levels of killing remained almost unchanged in two patients (nos 5 and 8), while there was a considerable decrease in the cytotoxic capacity of PBL from the remaining three patients (nos 2, 3 and 6) after rIL2 activation. Cytotoxicity against MPCa increased 2–20-fold in six patients (nos 1, 4–8), remained stable in one (no. 2) and decreased in one (no. 3) (Table I).

The use of a combination of rIL2 and rTNF-alpha for activation resulted in the development of higher cytotoxic capacity by PBL from all patients tested compared to their non-activated or rIL2-activated counterparts. In one instance where a high level of killing was already achieved using rIL2 alone (patient no. 2), the level of specific killing did not change significantly by the addition of rTNF-alpha. The effect of rTNF-alpha was most pronounced on the PBL which did not develop high levels of killing due to rIL2 activation (Table I).

*Development of cytostatic activity of PBL treated with rIL2 and a combination of rIL2 and rTNF-alpha*

On completion of the cytotoxicity assays the capacity of the effector cells to inhibit the growth of the surviving tumour target cells was examined. The results in Table II demonstrate that there was no correlation between the levels of cytotoxicity and cytostasis achieved by the untreated PBL. In spite of reasonable levels of natural killer activity in all test samples, cytostatic activity was present only in two against the K562 targets and in five against MPCa targets. Activation with rIL2 generated some cytostatic activity against K562 in all but two PBL samples. In these two samples (nos 3 and 7) the activated cells developed the capacity to stimulate the growth of K562 in spite of the fact that in patient no. 7 the cytotoxic activity of rIL2-activated LAK cells against this tumour was over 80%. rIL2 activation of PBL from patient no. 3 was detrimental to both cytotoxic and cytostatic responses.

The cytostatic activity against MPCa targets of all PBL samples increased 2–4-fold by rIL2 activation. Some samples (nos 4, 5 and 7), which had a pronounced stimulatory effect on these tumour cells, also became strongly inhibitory (Table II). The addition of rTNF-alpha to the activation cultures of the effector cells did not make a significant difference to the improved levels of cytostasis against MPCa achieved by PBL activated with rIL2 alone. Cytostasis against K562, however, was greatly improved when the lymphocytes were activated with rIL2 and rTNF-alpha. The detrimental effects of rIL2 activation on the cytostatic capacity of lymphocytes from patients 3 and 7 were reversed with the additional use of rTNF-alpha and the cytostatic capacity of lymphocytes from the remaining patients increased 2–6-fold (Table II).

**Table I** Cytotoxic activity of PBL and LAK cells from eight randomly selected cancer patients against two tumour cell lines

| | *Treatment of peripheral blood lymphocytes (% specific $^{51}$Cr-release from targets at effector to target ratios)* | | | | | | | | | | | |
|---|---|---|---|---|---|---|---|---|---|---|---|---|
| | *Nil* | | | | *rIL2* | | | | *rIL2+rTNF-α* | | | |
| | *K562* | | *MPCa* | | *K562* | | *MPCa* | | *K562* | | *MPCa* | |
| Patient no. | *5:1* | *2.5:1* | *5:1* | *2.5:1* | *5:1* | *2.5:1* | *5:1* | *2.5:1* | *5:1* | *2.5:1* | *5:1* | *2.5:1* |
| 1 | 36.2 | 24.2 | 16.0 | 9.4 | 64.6 | 40.1 | 33.0 | 25.5 | 76.3 | 65.4 | 64.7 | 65.1 |
| 2 | 21.1 | 16.5 | 13.4 | 10.1 | 15.0 | 8.6 | 13.4 | 11.0 | 49.6 | 33.1 | 50.3 | 38.2 |
| 3 | 19.9 | 9.7 | 6.9 | 4.9 | 6.0 | 4.0 | 3.1 | 5.5 | 42.3 | 24.6 | 50.6 | 37.4 |
| 4 | 16.9 | 18.3 | 9.6 | 10.1 | 55.2 | 30.7 | 31.1 | 21.6 | 66.4 | 42.0 | 58.5 | 41.1 |
| 5 | 48.8 | 26.3 | 17.5 | 9.7 | 40.6 | 26.0 | 33.0 | 23.8 | 62.0 | 44.3 | 61.7 | 48.6 |
| 6 | 25.8 | 19.3 | 8.2 | 4.9 | 18.5 | 9.2 | 13.9 | 8.2 | 68.8 | 54.8 | 64.7 | 47.1 |
| 7 | 52.4 | 19.1 | 10.4 | 7.0 | 82.8 | 64.7 | 54.0 | 36.5 | 76.8 | 71.6 | 78.5 | 73.3 |
| 8 | 21.5 | 9.2 | −1.2 | 2.6 | 22.7 | 13.0 | 21.5 | 16.4 | 76.9 | 56.9 | 62.0 | 54.3 |

**Table II** Cytostatic activity of untreated PBL and LAK cells from eight randomly selected cancer patients against two tumour cell lines

| | *Treatment of peripheral blood lymphocytes (% inhibition of $^{3}$H-thymidine uptake by targets at effector to target ratios)* | | | | | | | | | | | |
|---|---|---|---|---|---|---|---|---|---|---|---|---|
| | *Nil* | | | | *rIL2* | | | | *rIL2+rTNF-α* | | | |
| | *K562* | | *MPCa* | | *K562* | | *MPCa* | | *K562* | | *MPCa* | |
| *Patient no.* | *5:1* | *2.5:1* | *5:1* | *2.5:1* | *5:1* | *2.5:1* | *5:1* | *2.5:1* | *5:1* | *2.5:1* | *5:1* | *2.5:1* |
| 1 | −1.6 | 12.2 | 10.3 | 10.1 | 14.1 | 8.4 | 29.7 | 34.0 | 22.7 | 38.4 | 46.6 | 43.0 |
| 2 | −2.4 | 1.1 | 33.7 | 23.6 | 2.9 | 11.5 | 15.3 | 38.8 | 17.9 | 33.0 | 32.8 | 48.1 |
| 3 | −11.7 | 9.1 | 3.4 | 24.2 | −47.8 | 23.3 | 24.4 | 21.7 | 26.0 | 40.7 | 31.1 | 58.4 |
| 4 | −11.7 | −18.3 | −35.1 | −1.0 | 8.3 | 27.7 | 35.0 | 30.0 | 30.5 | 29.6 | 29.8 | 38.2 |
| 5 | −5.3 | 19.6 | −12.9 | 12.2 | 15.7 | 4.0 | 38.0 | 44.2 | 30.8 | 17.8 | 38.4 | 62.6 |
| 6 | 21.9 | 10.2 | 14.5 | 21.2 | 33.4 | 14.0 | 37.2 | 29.4 | 36.2 | 32.1 | 45.1 | 39.3 |
| 7 | 4.7 | −3.0 | −11.0 | 2.8 | −13.1 | 10.5 | 24.5 | 25.0 | 13.0 | 39.8 | 33.0 | 49.0 |
| 8 | 18.1 | 29.5 | 12.3 | 19.0 | 5.7 | 32.8 | 29.7 | 28.8 | 35.9 | 37.2 | 43.6 | 39.0 |

**Table III** Cytotoxic activity of untreated splenic lymphocytes and splenic LAK cells from four cancer patients against two tumour cell lines

| *Treatment of splenic lymphocytes* | *% specific $^{51}$Cr-release from target cells at effector to target ratios* | | | | | | | |
|---|---|---|---|---|---|---|---|---|
| | *K562* | | *MPCa* | | *K562* | | *MPCa* | |
| | *5:1* | *2.5:1* | *5:1* | *2.5:1* | *5:1* | *2.5:1* | *5:1* | *2.5:1* |
| | *Patient 1* | | | | *Patient 2* | | | |
| Nil | 20.6 | 13.2 | 23.0 | 22.6 | 27.0 | 18.5 | 18.5 | 8.8 |
| rIL2 | 45.5 | 13.7 | 50.7 | 46.5 | 46.5 | 30.6 | 30.1 | 16.0 |
| rIL2+rTNF-α | 81.4 | 65.0 | 37.0 | 23.7 | 55.5 | 36.0 | 33.7 | 27.4 |
| | *Patient 3* | | | | *Patient 4* | | | |
| Nil | 45.0 | 28.8 | 52.3 | 38.7 | 59.3 | 35.0 | 67.0 | 43.1 |
| rIL2 | 94.6 | 85.5 | 71.9 | 74.5 | 90.7 | 68.2 | 79.8 | 65.5 |
| rIL2+rTNF-α | 85.6 | 79.0 | 70.3 | 45.0 | 85.7 | 71.3 | 77.3 | 64.7 |

*Generation of cytotoxic activity in splenic lymphocytes using rIL2 and rTNF-alpha*

The potential development of LAK activity from splenocytes was examined using cells from four spleens removed during surgical resection for gastro-oesophageal and pancreatic carcinoma. Untreated splenocytes from two patients had low cytotoxic activity against K562 and MPCa ranging from 18.5 to 27%, while splenocytes from the remaining two patients achieved levels of killing ranging from 45 to 67% (Table III). Activation with rIL2 increased the cytotoxic capacity of the splenocytes from the former two patients to the range 30–45% and the latter to 72–95%. Preincubation of the splenocytes with additional rTNF-alpha did not confer further improvement to the latter group but significantly improved the cytotoxic potential of the former group with levels of killing within the range 33–81% (Table III).

*Cytotoxicity of LAK cells against NK-resistant K562 targets*

K562 target cells which survived cytotoxicity by NK cells in a preparative assay were allowed to grow in culture and were used as target cells to test the cytotoxic capacity of LAK cells activated with rIL2 or a combination of rIL2 and rTNF-alpha. Results in Table IV demonstrate that one cycle of NK selection of K562 leukaemic cells increases the resistance of these cells to a subsequent NK cell attack by 60–73% at effector to target cell ratios of 5:1 and 2.5:1. The resistance to killing by rIL2 activated lymphocytes was considerably less and in the range 12.5–42% in two experiments, while the least resistance, ranging from 3.4–24.6%, was presented to killer cells activated with a combination of rIL2 and rTNF-alpha.

### Discussion

The results demonstrate that there is no correlation between the levels of NK cell killing of K562 and MPCa by the PBL of the different individuals examined. There are also differential changes in activity against these two targets in response to rIL2 activation (Table I). This would suggest that different lytic mechanisms may be operating against these two targets, possibly via different effector cell populations. There is also no correlation between the cytotoxic capacity of the PBL and their potential to inhibit the proliferation of tumour target cells. This indicates that different mechanisms are involved in cytotoxicity and cytostasis, which is another multi-effector function mediated by several lymphocyte subsets (Matossian-Rogers & Taidi, 1983). In the present experiments whole peripheral blood mononuclear cells containing monocytes were used and these can potentially contribute to cytotoxic and cytostatic reactions; we have, however, used these terms operationally to define a function rather than a particular cell type. Successful immunotherapeutic procedures must aim to enhance both these effector systems.

Treatment of PBL with rIL2 did not improve the cytotoxic potential of all patients' lymphocytes. In fact the cytotoxic capacity of five out of eight samples remained either unaltered or deteriorated after 3 days' culture with rIL2 (Table I). The generation of cytotoxic capacity in IL2-stimulated PBL is not only dependent on IL2 but also on the interaction of other lymphokines and cytokines produced by the stimulated cells. Large variations in the production of interferon-gamma, TNF-beta and TNF-alpha by IL2 stimulated PBL from different donors have been reported (Meager *et al.*, 1987).

Recent experiments demonstrate that the extent of activation of NK cells by exposure to IL2 is variable and donor-dependent (Titus *et al.*, 1987). This is possibly due to the different levels of synergising cytokines produced in response to IL2 by different donors. The results of Meager *et al.* (1987) demonstrate that production of TNF-beta by PBL was only weakly enhanced by rIL2 stimulation and in some donors TNF-alpha levels of rIL2-stimulated cultures

**Table IV** Cytotoxicity of rIL2– and rIL2+rTNF-α activated LAK cells against natural killer cell selected (NKCS) K562 target cells

| Exp. no. | Target cells | Nil 5:1 | Nil 2.5:1 | rIL2 5:1 | rIL2 2.5:1 | rIL2+rTNF-α 5:1 | rIL2+rTNF-α 2.5:1 |
|---|---|---|---|---|---|---|---|
| | | *Treatment of effector lymphocytes (% specific $^{51}$Cr-release from targets at effector to target ratios)* | | | | | |
| 1 | K562 | 27.3 | 15.0 | 38.0 | 20.0 | 48.6 | 24.8 |
| | K562-NKCS | 10.9 (60.1)[a] | 4.4 (70.7) | 22.0 (42.2) | 11.9 (40.5) | 39.3 (19.2) | 18.7 (24.6) |
| 2 | K562 | 24.5 | 12.6 | 69.1 | 45.6 | 71.4 | 48.9 |
| | K562-NKCS | 6.7 (72.7) | 3.4 (73.1) | 52.9 (23.5) | 39.9 (12.5) | 68.3 (4.4) | 47.1 (3.7) |

[a]Percentage resistance to killing calculated according to the formula:

$$100-\left[\frac{\%\text{ specific release from K562-NKCS}}{\%\text{ specific release from K562}}\right]\times 100.$$

were lower than those of unstimulated cultures. TNF-alpha has numerous immunomodulatory effects which include the enhancement of cytotoxicity in monocytes (Philip & Epstein, 1986), the induction of IL2 receptors on CD16+ large granular lymphocytes (Øtensen *et al.*, 1987) and IL1 production by human monocytes and endothelial cells (Dinarello *et al.*, 1986).

In our experiments the incubation of patients' PBL with rIL2 and additional rTNF-alpha endowed the non- or low-responders to rIL2 with high levels of cytotoxic capacity (Table I). PBL which showed a strong response to rIL2 were not affected significantly by additional exposure to rTNF-alpha. Culture of PBL with rTNF-alpha alone did not induce strong cytotoxic capacity in any of the samples tested (data not shown). Apart from the increased expression of receptors for IL2 induced by TNF-alpha the induction of peripheral blood mononuclear cells to secrete IL1 may have a role in the enhanced killing capacity of the patients' lymphocytes. Cytotoxic activity of large granular lymphocytes has been shown to be closely related to IL1 production. Monocytes from patients with malignant disease have been shown to be defective in IL1 production and have depressed NK cell activity (Son *et al.*, 1982; Herman *et al.*, 1984). Herman *et al.* (1985) demonstrated that K562 target cells treated with IL1 bound greater numbers of LGL than untreated target cells and defective cytotoxicity of LGL from patients with hepatocellular carcinoma could be corrected by treating the target K562 cells with IL1.

TNF-alpha used in combination with rIL2 not only improved the cytotoxic capacity of LAK cells but also their capacity to prevent the proliferation of the target cells (Table II). Cytotoxicity of tumour targets even at high effector to target cell ratios is rarely complete and surviving tumour cells can proliferate. Our results demonstrate that K562 target cells which survived NK cell cytotoxicity and grew out (K562 NKCS) are 60–73% more resistant to NK cells than the parent K562 population (Table IV). Similar results were obtained with K562, which survived killing by LAK cells (data not shown). It is thus important that LAK cells exert a cytostatic effect on tumour cells as well as being cytolytic. rTNF-alpha used in addition to rIL2 to generate LAK cell activity increased the cytostatic activity against K562 of all samples of PBL tested. Suzuki *et al.* (1987) demonstrated that tumour cells remained dormant in the peritoneal cavity of immune mice due to the cytostatic action of host cells induced by the synergistic action of IFN-gamma and TNF-alpha.

It appears that effector cells activated by the use of rIL2 and rTNF-alpha are more aggressive killers and also cytostatic. Phenotypic analysis of comparable cultures of LAK cells activated by rIL2 in combination with rTNF-alpha showed no differences in these cell populations (data not shown). There were also no differences in cell numbers, indicating that there was no differential outgrowth of cell subsets (data not shown). The enhanced killing capacity of rIL2+rTNF-alpha-LAK cells may be due to a combination of factors, such as the induction of IL1 production, the increased expression of IL2 receptors and other immunoregulatory effects of TNF-alpha on lymphocytes and monocytes, such as the induction of the synthesis of novel lytic or cytostatic proteins (Ruggiero *et al.*, 1987). Another possibility is the recognition of a broader spectrum of targets on tumour cells by rIL2+rTNF-alpha-LAK cells than by those activated by rIL2 alone. K562 target cells which survived killing by NK cells expressed a decreasing order of resistance to subsequent killing by NK, rIL2-LAK and rIL2+rTNF-alpha-LAK cells (Table IV). One can postulate that rIL2-LAK cells recognise NK cell targets as well as another set of determinants while rIL2+rTNF-alpha-LAK cells recognise both these sets of targets and also a third family of target structures on tumour cells. Whatever the mechanism of the improved performance of rIL2+rTNF-alpha-LAK cells it is apparent that the additional use of rTNF-alpha in the preparation of LAK cells from PBL or splenocytes could improve the success rate of LAK cell immunotherapy.

### References

BERTOLINI, D.R., NEDWIN, G.E., BRINGMAN, T.S., SMITH, D.D. & MUNDY, G.R. (1986). Stimulation of bone resorption and inhibition of bone formation *in vitro* by human tumour necrosis factors. *Nature*, **319**, 516.

BENTLER, B. & CERAMI, A. (1986). Cachectin and tumour necrosis factor as two sides of the same biological coin. *Nature*, **320**, 584.

DAYER, J.M., BEUTLER, B. & CERAMI, A. (1985). Cachectin/tumour necrosis factor stimulates collagenase and prostaglandin $E_2$ production by human synovial cells and dermal fibroblasts. *J. Exp. Med.*, **162**, 2163.

DINARELLO, C.A., CANNON, J.G., WOLF, S.M. and 6 others (1986). Tumour necrosis factor (cachectin) is an endogenous pyrogen and induces production of interleukin 1. *J. Exp. Med.*, **163**, 1433.

GRIMM, E., MAZUMDER, A., ZHANG, H. & ROSENBERG, S. (1982). Lymphokine-activated killer phenomenon: lysis of natural killer-resistant fresh solid tumour cells by interleukin-2 activated autologous human peripheral blood lymphocytes. *J. Exp. Med.*, **155**, 1823.

GRIMM, E., ROBLE, R., ROTH, J. and 4 others (1983). Lymphokine-activated killer cell phenomenon: III. Evidence that IL-2 is sufficient for direct activation of peripheral blood lymphocytes into lymphokine-activated killer cells. *J. Exp. Med.*, **158**, 1356.

HERMAN, J.M., DINARELLO, C.A., KEW, M.C. & ROBSON, A.R. (1985). The role of interleukin 1 (IL1) in tumour-NK cell interactions: correlation of defective NK cell activity in cancer patients by treating target cells with IL1. *J. Immunol.*, **135**, 2882.

HERMAN, J.M., KEW, C. & ROBSON, A.R. (1984). Defective interleukin-1 production of monocytes from patients with malignant disease. Interferon increases IL1 production. *Cancer Immunol. Immunother.*, **16**, 182.

MATOSSIAN-ROGERS, A. & TAIDI, B. (1983). Characterization of cytostatic effector lymphocytes during the development of a syngeneic lymphosarcoma in C3H mice: use of monoclonal reagents to identify T-cell subsets. *Cell. Immunol.*, **82**, 292.

MEAGER, A., PARTI, S., LEUNG, H. and 4 others (1987). A two-site sandwich immuno-radiometric assay of human lymphotoxin with monoclonal antibodies and its applications. *J. Immunol. Methods*, **104**, 31.

MUUL, L.M., DIRECTOR, G.P., HYATT, C. & ROSENBERG, S. (1986). Large scale production of human lymphokine activated killer cells for use in adoptive immunotherapy. *J. Immunol. Methods*, **88**, 265.

NAWROTH, P.P. & STERN, D.M. (1986). Modulation of endothelial cell hemostatic properties by tumour necrosis factor. *J. Exp. Med.*, **163**, 740.

OCHOA, C. A., GRANO, G., ALTER, B.J., SONDEL, P.M. & BACH, F.H. (1987). Long-term growth of lymphokine-activated killer (LAK) cells: role of anti-CD3, beta-IL1, interferon-alpha and -beta. *J. Immunol.*, **138**, 2728.

ØTENSEN, M.E., THIELE, D.L. & LIPSKY, P.E. (1987). Tumour necrosis factor-alpha enhances cytolytic activity of human natural killer cells. *J. Immunol.*, **138**, 4185.

PALLADINO, M.A. JR, SHALABY, M.R., KRAMER, S.M. and 9 others (1987). Characterisation of the anti-tumour activities of human tumour necrosis factor-alpha and the comparison with other cytokines: induction of tumour specific immunity. *J. Immunol.*, **138**, 4023.

PHILIP, R. & EPSTEIN, L.B. (1986). Tumour necrosis factor as immunomodulator of monocyte cytotoxicity induced by itself, gamma-interferon and interleukin 1. *Nature*, **326**, 86.

ROSENBERG, S., LOTZE, M., MUUL, M. and 10 others (1985). Observations on the systemic administration of autologous lymphokine-activated killer cells and recombinant interleukin-2 to patients with metastatic cancer. *N. Engl. J. Med.*, **313**, 1485.

ROSENBERG, S., LOTZE, M., MUUL, M. and 10 others (1987). A progress report on the treatment of 157 patients with advanced cancer using lymphokine-activated killer cells and interleukin-2 or high-dose interleukin-2 alone. *N. Engl. J. Med.*, **316**, 889.

RUGGIERO, V., LATHAM, K. & BAGLIANI, C. (1987). Cytostatic and cytotoxic activity of tumour necrosis factor on human cancer cells. *J. Immunol.*, **138**, 2711.

SUGARMAN, B.J., AGGARWAL, B.B., HASS, P.E., FIGARI, I.S., PALLADINO, M.A. & SHEPARD, H.M. (1985). Recombinant human tumour necrosis factor-alpha: effects on proliferation of normal and transformed cells *in vitro*. *Science*, **230**, 943.

SON, K., KEW, M.C. & ROBSON, A.R. (1982). Depressed natural killer cell activity in patients with hepatocellular carcinoma. *In vitro* effects of interferon and levimasole. *Cancer*, **50**, 2820.

SUZUKI, Y., LIU, C.-M., CHEN, L., BEN-NATHAN, D. & WHEELOCK, E.F. (1987). Immune regulation of the L5178Y murine tumour-dormant state. II. Interferon-gamma requires tumour necrosis factor to restrain tumour cell growth in peritoneal cell cultures from tumour-dormant mice. *J. Immunol.*, **139**, 3146.

TORTI, F.M., DIECKMANN, B., BEUTLER, B., CERAMI, A. & RINGOLD, G.M. (1985). A macrophage factor inhibits adipocyte gene expression: an *in vitro* model of cachexia. *Science*, **229**, 867.

TITUS, J.A., PEREZ, P., KAUBISCH, A., GARRIDO, M.A. & SEGAL, D.M. (1987). Human K/natural killer cells targeted with hetero-cross-linked antibodies specifically lyse tumour cells *in vitro* and prevent tumour growth *in vivo*. *J. Immunol.*, **139**, 3153.

WILLIAMSON, B.D., CARSWELL, E.A., RUBIN, B.Y., PRENDERGAST, Y.S. & OLD, L.J. (1983). Human tumour necrosis factor produced by human B-cell lines: synergistic cytotoxic interaction with human interferon. *Proc. Natl Acad. Sci. USA*, **80**, 5397.